# Changes in the facial soft tissue profile after maxillary orthognathic surgery

**DOI:** 10.1007/s00056-021-00294-2

**Published:** 2021-04-21

**Authors:** S. Rupperti, P. Winterhalder, S. Krennmair, S. Holberg, C. Holberg, G. Mast, I. Rudzki

**Affiliations:** 1 Department of Orthodontics, Ludwig-Maximilians University of Munich, Munich, Germany; 2 Department of Oral and Maxillofacial Surgery, RWTH Aachen, Aachen, Germany; 3 Department of Oral and Maxillofacial Surgery, Ludwig-Maximilians University of Munich, Munich, Germany

**Keywords:** Cephalometry, Maxillary osteotomy, Esthetics, Orthognathic surgical procedures, Treatment outcome, prediction, Kephalometrie, Maxilläre Osteotomie, Ästhetik, Kieferorthopädische chirurgische Eingriffe, Behandlungsergebnis, Vorhersage

## Abstract

**Objectives:**

To compare the changes of the soft tissue profile in relation to the displacement of the underlying hard structures in maxillary orthognathic surgery and to contribute to the esthetic prediction of the facial profile after surgical procedures.

**Materials and methods:**

We analyzed the sagittal changes in the facial soft tissue profile related to surgical changes in skeletal structures after maxillary osteotomy in a retrospective study. The study sample comprised 115 adult patients between the ages of 18–50 years who had undergone maxillary orthognathic surgery and interdisciplinary orthodontic treatment at the Department of Orthodontics, Ludwig-Maximilians University of Munich, Germany. LeFort I osteotomy cases in both maxillary monognathic and bignathic osteotomy procedures were included. All subjects had received rigid fixation. A cephalometric analysis of presurgical and postsurgical cephalograms was performed and the correlations between hard tissue and soft tissue change ratios were evaluated using a bivariate linear regression analysis. A vertical line through the landmark sella (S) perpendicular to the nasion-sella line (NSL) served as the reference plane.

**Results:**

The subnasale (Sn) followed the A point (A) by 57%, the soft tissue A point (A′) followed the A point (A) by 73% and the upper lip, represented by the landmark labrale superius (Ls) followed the upper incisor (Is) by 73%; all three in a linear correlation with a mean prediction error of nearly 2 mm.

**Conclusion:**

The scatterplots show a linear correlation with a wide spread for all three pairs of reference points. The wide spread and the high prediction error of almost 2 mm indicate low predictability of the expected lip position and Sn.

## Abstract (de)

**Ziele:**

Die Veränderungen des Weichgewebeprofils in Relation zur Verlagerung der darunter liegenden Hartgewebe durch maxilläre orthognathe Chirurgie zu vergleichen und einen Beitrag zur ästhetischen Prognose des Gesichtsprofils nach chirurgischen Maßnahmen zu leisten.

**Material und Methode:**

In einer retrospektiven Studie analysierten wir die sagittalen Veränderungen des fazialen Weichgewebeprofils in Beziehung zu den chirurgischen Veränderungen der skelettalen Strukturen nach maxillärer Osteotomie. Die Studienprobe besteht aus 115 erwachsenen Patienten im Alter von 18–50 Jahren, die sich interdisziplinär maxillärer orthognather Chirurgie und kieferorthopädischer Therapie an der Poliklinik für Kieferorthopädie der Ludwig-Maximilians-Universität München unterzogen hatten. LeFort-I-Osteotomie-Fälle sowohl maxillärer monognather als auch bignather Osteotomieverfahren wurden in die Studie aufgenommen. Alle Patienten hatten eine starre Fixierung erhalten. Es wurde eine kephalometrische Analyse von präoperativen und postoperativen Fernröntgenseitenbildern durchgeführt, die Korrelationen zwischen Hart- und Weichgewebeveränderungen wurden mittels einer bivariaten linearen Regressionsanalyse ausgewertet. Als Referenzebene diente eine vertikale Linie durch den Referenzpunkt Sella (S), rechtwinklig zur Nasion-Sella-Linie (NSL).

**Ergebnisse:**

Der Punkt Subnasale (Sn) folgte dem A‑Punkt (A) um 57 %, der Weichgewebe-A-Punkt (A′) folgte dem A‑Punkt (A) um 73 %, und die Oberlippe, repräsentiert durch den Referenzpunkt Labrale superius (Ls), folgte dem Inzision superius (Is) um 73 %, alle 3 in einer linearen Korrelation bei einer mittleren Abweichung von fast 2 mm.

**Fazit:**

Die Punktdiagramme zeigen eine lineare Korrelation mit einer breiten Streuung aller 3 Referenzpunktpaare. Die breite Streuung und die hohe mittlere Abweichung von fast 2 mm lassen auf eine schwache Vorhersagbarkeit der zu erwartenden Position von Oberlippe und Sn schließen.

## Introduction

Orthognathic surgery is the treatment of choice for severe skeletal dysgnathia and dentofacial deformities. The surgical discipline, which began in 1849 in the USA, has evolved progressively in central Europe since the 1950s and today is commonly applied [29]. Although most patients benefit from surgery [20, 23], up to 22% are dissatisfied with the esthetic result [10]. The esthetic improvement of the facial profile very much affects the social and psychological well-being of the patient [3, 9, 10]. Prediction of the soft tissue profile is of great importance in treatment planning and patient motivation [16].

The first studies analyzing the soft tissue profile change after orthognathic surgery were published in the 1970s with the purpose to acquire more information that can be used as an esthetic guide in orthognathic surgery [18, 21, 25].

We included 115 maxillary LeFort I osteotomy cases in monognathic and bignathic procedures in this study. Our target was a contribution to the facial profile prediction after maxillary orthognathic surgery by providing profound data of the soft to hard tissue dependencies.

## Materials and methods

In this retrospective study, we analyzed 115 patients on whom interdisciplinary orthodontic and orthognathic surgical treatment had been performed at the Department of Orthodontics and the Department of Oral and Maxillofacial Surgery, Ludwig-Maximilians University of Munich, Germany (Table 1).

**Table 1:** Descriptive statistics of the 115 patients Deskriptive Statistik der 115 Patienten

	Men	Women
Patients total (*n*)	54	61
Maxillary surgery (*n*)	15	20
Bignathic surgery (*n*)	39	41
Class II	16	27
Class III	38	34
Mean age at surgery (years)	27	28
Standard deviation of age (years)	6	7
Minimum age (years)	18	18
Maximum age (years)	45	50

The sample consists of 43 class II and 72 class III patients, all interbasal open O1 and N1 types with divergent inclination of the skeletal bases, with posterior inclination of the mandible and anterior inclination of the maxilla in various manifestations.

On all 115 subjects LeFort I osteotomy with posterior maxillary impaction had been performed, 35 in monognathic and 80 in bignathic procedures. All 115 subjects received rigid fixation. For both females and males the minimum age was 18 in order to avoid errors caused by the influence of growth. A history of prior maxillofacial surgery, wire fixation, trauma, clefts and craniofacial syndromes were further exclusion criteria.

The sample size was calculated for a power of 0.8 at a significance level of 0.05 with Altman’s nomogram [2]. As 15 patients were excluded because of unconfident landmark identification caused by insufficient radiograph quality, the study had a power of 0.78 at a significance level of 0.05.

For each subject a preoperative and a postoperative radiograph, taken at least 6 months after surgery, was selected. A Canon EOS 5D digital camera with a Canon compact-macro EF 50 mm, f 1:2.5 lens (Canon Inc., Tokio, Japan) on a “copylizer eVision exe.cutive” camera stand (Kaiser Fototechnik GmbH &Co. KG, Buchen, Germany) was used for the digitization process of the radiographs. One of the authors (S. R.) conducted a cephalometric analysis based on the method of Segner/Hasund [28] with the software DiagnoseFix 12.2006 (Dr. Jörg Wingberg, Buchholz, Germany).

**Fig. 1:**
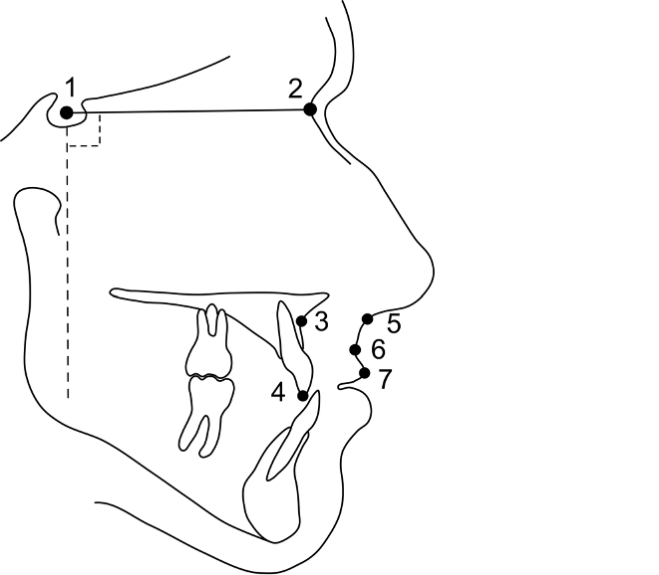
Landmarks and reference lines used in this study: *1* sella; *2* nasion; *3* A point; *4* incision superius; *5* subnasale; *6* soft tissue A point; *7* labrale superius In der Studie verwendete Referenzpunkte und -linien. *1* Sella; *2* Nasion; *3* A-Punkt; *4* Incision superius; *5* Subnasale; *6* Weichgewebe-A-Punkt; *7* Labrale superius

On the basis of the analysis of Lines and Steinhauser [18] and Legan and Burstone [16] our cephalometric analysis was reduced to the examination of these relevant landmarks:

The soft tissue landmarks Sn (subnasale), A′ (soft tissue A) and Ls (labrale superius) and the corresponding hard tissue landmarks A (A point) and Is (incision superius) (Fig. 1). A coordinate system was designed to assess the surgical movement in the sagittal direction. NSL (nasion-sella line) was used as the x‑axis, while the y‑axis was constructed as a line through the landmark sella, perpendicular to the NSL. This y‑axis served as the vertical reference line for the examined landmarks.

We quantified the distance from the vertical reference line to each soft and hard tissue point and calculated the difference of the postsurgical minus the presurgical values.

The correlations between the shift in soft tissue landmarks (∆Sn, ∆A′ and ∆Ls) and the shift in the corresponding hard tissue landmarks (∆A and ∆Is) were then statistically analyzed. We conducted a bivariate linear regression analysis to determine the soft tissue profile changes related to surgical movement of the underlying hard tissue structures (R 3.1.2, R Foundation for Statistical Computing, Vienna, Austria).

## Results

### Soft tissue A point

**Fig. 2:**
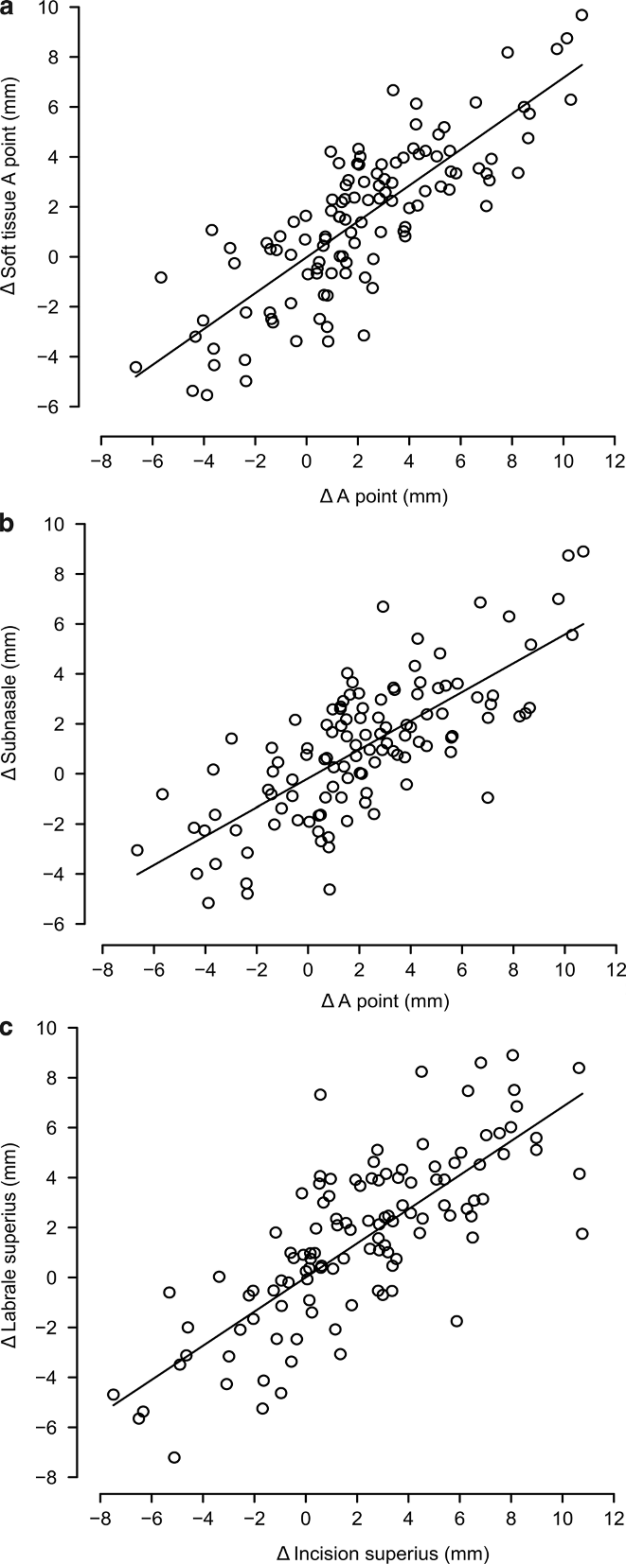
Scatterplot and prediction model of sagittal changes for soft tissue A point (**a**), subnasale (**b**), and labrale superius (**c**) Streudiagramm und Vorhersagemodell der sagittalen Veränderungen für Weichgewebe-A-Punkt (**a**), Subnasale (**b**) und Labrale superius (**c**)

Soft tissue A point changed minimum −5.5 mm and maximum 9.7 mm, while hard tissue A point changed minimum −6.6 mm and maximum 10.7 mm. The Shapiro–Wilk test showed normal distribution for displacements both in soft tissue A point (*p* = 0.53) and in hard tissue A point (*p* = 0.35). The Pearson correlation coefficient for soft tissue A point and hard tissue A point was 0.83 (Fig. 2a). The model to predict the change of soft tissue A point was *soft tissue A point* *=* 0.73 × *hard tissue A point* with a coefficient of determination of r^2^ = 0.69 and a standard error of the estimate of 1.7 mm.

### Subnasale

Displacements of subnasale ranged from minimum −5.2 mm to maximum 8.9 mm and were normally distributed according to the Shapiro–Wilk test (*p* = 0.42). The Pearson correlation coefficient for subnasale and hard tissue A point was 0.76 (Fig. 2b). Displacements in subnasale were predicted by the model *Subnasale* = 0.57 × *hard tissue A point.* The coefficient of determination was r^2^ = 0.58 with a standard error of the estimate of 1.8 mm.

### Labrale superius

Labrale superius changed minimum −7.2 mm and maximum 8.9 mm, whereas incision superius changed minimum −7.5 mm and maximum 10.7 mm. Normal distribution of the displacements was indicated by a Shapiro–Wilk test for labrale superius (*p* = 0.47) and incision superius (*p* = 0.73). Pearson’s correlation coefficient for labrale superius and incision superius was 0.81 (Fig. 2c). The prediction model of displacements for labrale superius was *Labrale superius* = 0.73 × *Incision superius* with a coefficient of determination of r^2^ = 0.66 and a standard error of the estimate of 1.9 mm.

**Fig. 3:**
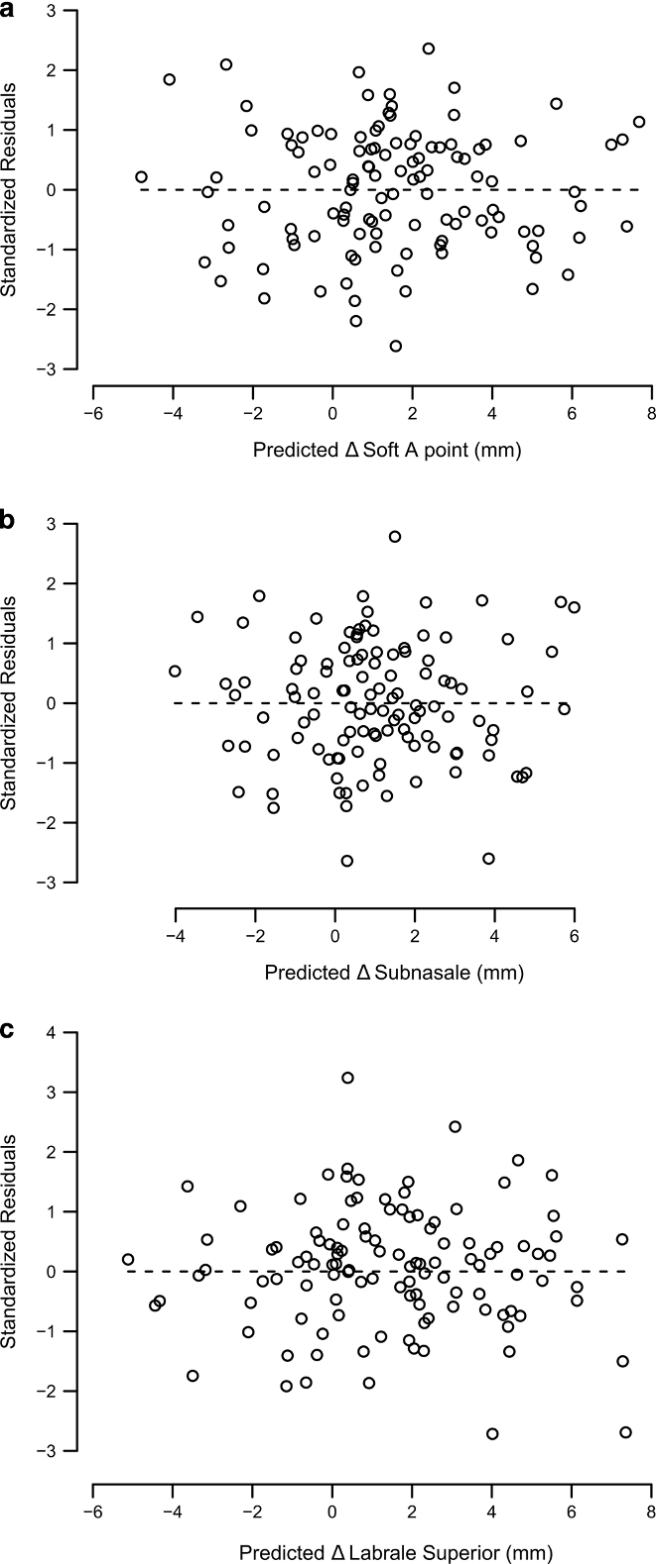
Residual plots for predicted sagittal changes in soft tissue A point (**a**), subnasale (**b**), and labrale superius (**c**) Residuenplots für die prognostizierten sagittalen Veränderungen von Weichgewebe-A-Punkt (**a**), Subnasale (**b**) und Labrale superius (**c**)

The scatterplots (Fig. 2) show a linear correlation between each pair of landmarks, but with a wide spread for all three pairs. The residuals of the prediction models were symmetrically distributed without any recognizable pattern that would indicate another additional prediction variable (Fig. 3).

## Discussion

Our results in this study for A′/A (73%), Sn/A (57%) and Ls/Is (73%) correspond to many other studies [4–6, 17, 18, 24]. Some authors found results equal to ours by using slightly different methods, like for example different reference lines [4, 24, 30]. But there also exists a high variation of different results in literature [7, 8, 14, 15, 27]. The reasons that could be responsible for this high variance might be tonicity, posture, muscle pull and difficulty adopting a relaxed lip position during cephalogram exposure [22]. Another reason might as well be that the soft tissues follow the maxillary hard tissue structures in a relationship not as close as in the mandible [26] because the soft tissue of the upper lip is firmly connected to the base of the nose [18]. Furthermore, the lower lip also has an influence on the position of the upper lip. A surgical change of the position of the maxilla leads to a change of the position of the mandible and of the lower lip, both in bignathic and monognathic surgery.

It is apparent that maxillary soft tissue depends on a complexity of functional and anatomical influences, which might be the reason for our rather high prediction error of about 2 mm, as well as the high variety of results in literature.

Although landmark localization on lateral cephalograms may be impaired by distortion or magnification [11], this two-dimensional method offers high reliability [1, 12].

Three-dimensional technologies can improve diagnostics providing a highly accurate reproduction of the facial morphology and even a very precise automatic cephalometry with exact landmark detection [13, 19]. However, our objective was to evaluate the ratio of the soft to hard tissue changes. The lateral cephalogram displays both hard and soft tissue structures in just one image, and that at a very low radiation exposure. Therefore, it was the medium of choice for our purpose. Furthermore, most clinicians simply do not have three-dimensional equipment, which still makes radiographs a widespread and valuable technique that should not be underestimated [26].

## Conclusions

In this retrospective study we revealed a linear correlation between each pair of soft and hard tissue landmarks. But at the same time all three measurements demonstrated a wide distribution of measurement values. This outcome and also the mean prediction error of about 2 mm prompts a cautious use of postsurgical predictions of the maxillary-related soft tissue profile changes.
